# The associations between healthy lifestyle scores, their long-term changes, and incident hypertension risk in adults: a prospective cohort study

**DOI:** 10.3389/fcvm.2025.1678746

**Published:** 2026-01-21

**Authors:** Ji Zhang, Tao Liu, Yanli Wu, Jie Zhou, Ling Li, Xuejiao Li, Wei Ji

**Affiliations:** Department of Chronic Disease Prevention and Control, Guizhou Center for Disease Control and Prevention, Guiyang, Guizhou, China

**Keywords:** cohort study, healthy lifestyle scores, hypertension, incidence, long-term changes

## Abstract

**Objective:**

Several modifiable healthy lifestyle factors have been demonstrated to exert an effect of blood pressure lowering. However, there remains limited evidence regarding the association between healthy lifestyle scores (HLS) and hypertension, particularly within the Chinese population. The present prospective cohort study was designed to systematically investigate the relationships between HLS, their long-term dynamic changes, and the incident risk of hypertension.

**Methods:**

A total of 3,743 participants aged ≥18 years who were free of hypertension at baseline were included in the study and followed up prospectively. The HLS was calculated based on the number of healthy lifestyle factors. The Cox proportional hazards regression model was employed to estimate hazard ratios (HRs) and corresponding 95% confidence intervals (95% CIs) for evaluating the associations between HLS, their long-term changes, and the risk of incident hypertension. In addition, a quantile regression (QR) approach was applied to assess the associations of HLS with blood pressure levels.

**Results:**

During a median follow-up period of 6.46 years, 857 cases of hypertension were identified. The HLS was significantly associated with a reduced risk of hypertension, with a 14% risk reduction observed (HR: 0.86, 95% CI: 0.80–0.92). Compared with the low HLS group (0–3), the HRs (95% CIs) for incident hypertension were 0.80 (95% CI: 0.66–0.97), 0.78 (95% CI: 0.64–0.95), and 0.61 (95% CI: 0.49–0.75) for the HLS groups with 4, 5, and ≥6, respectively (P for trend <0.001). In comparison with participants maintaining a HLS of 0–3, those maintaining a HLS of 4 (HR: 0.60, 95% CI: 0.43–0.85), 5 (HR: 0.47, 95% CI: 0.32–0.69), and ≥6 (HR: 0.34, 95% CI: 0.23–0.51) exhibited a significantly lower risk of incident hypertension (P for trend <0.001). QR showed a significant negative association between HLS and blood pressure levels.

**Conclusion:**

As the HLS increased, the risk of hypertension showed a gradual downward trend. Furthermore, long-term maintenance of a high HLS was associated with a reduced risk of hypertension. Our findings provide additional evidence from China supporting the necessity of sustaining healthy lifestyles across the life course.

## 1 Introduction

Hypertension is reported to be a major risk factor for cardiovascular disease (CVD) and other diseases (1). It is estimated that approximately 33% of the global population aged 30–79 years lives with high blood pressure, and the number of case has doubled in the last three decades, reaching approximately 1.3 billion worldwide in 2019 (2). In China, the burden of hypertension has increased as a result of urbanization and population aging (3). Despite the widespread efforts to prevent and treat hypertension, nearly 274 million Chinese adults aged 18–69 years are affected by the condition, and the blood pressure control rate remains low, with only 12.0% of cases under control (4). Hypertension is a global public health challenge that imposes substantial burden on both patients and healthcare systems, and therefore, it is critical to identify effective strategies for its primary prevention.

Studies have demonstrated that the development of hypertension is associated with genetic factors, lifestyle factors, as well as their interactions (5–7). To date, growing evidence indicates that lifestyle factors play an important role in hypertension pathogenesis. A great deal of research has independently confirmed that multiple dimensions of individual lifestyle factors, including diet (8), physical activity (9), body weight (10), sleeping behavior (11), tobacco use (12), and alcohol consumption (13), influence blood pressure levels. A prospective cohort from the UK Biobank study observed that adherence to a healthy lifestyle can partially counteract the genetic risk of elevated blood pressure and its associated consequences (14). Another cohort study from the China Kadoorie Biobank revealed that healthy lifestyles may significantly reduce the risk of CVD in adults (15). Consequently, lifestyle modification is recommended as a first-line strategy in all major hypertension management guidelines (16, 17).

Despite the fact that healthy lifestyles such as maintaining a healthy diet, engaging in regular physical activity, keeping weight under control, avoiding smoking, and reducing alcohol consumption have been reported to benefit hypertension prevention (18), there remains a relative paucity of research on their combined effects on hypertension. On account of the fact that multiple health-related lifestyle behaviors often coexist in individuals, it is essential to consider health lifestyle factors simultaneously to maximize public health impact. To our knowledge, a small number of prospective cohort studies have been conducted on this issue among the Chinese community population, especially pertaining to the associations between long-term dynamic changes in lifestyle behaviors and hypertension. Therefore, based on a prospective cohort study of adults from Southwest China with heterogeneity in their demographical characteristics and lifestyles and using the Cox proportional hazards regression model and quantile regression (QR) method, we assess the combined effects of a healthy lifestyle score (HLS), comprising the identified modifiable factors related to hypertension, on the incidence of hypertension and blood pressure levels. In addition, we further investigate the relationships between long-term changes (change from baseline to follow-up) in HLS and the risk of hypertension.

## 2 Methods

### 2.1 Study design and participants

**Figure 1:**
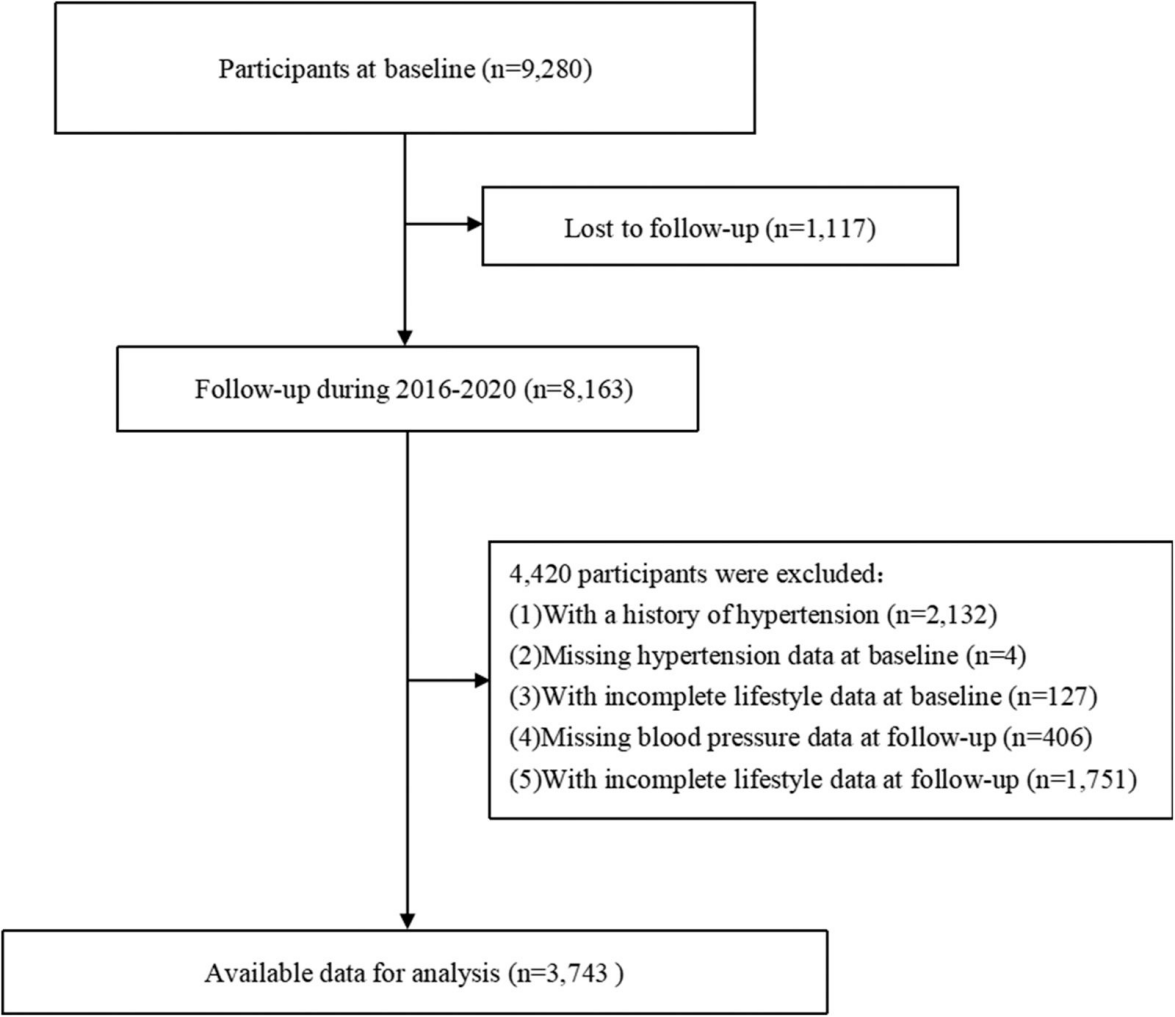
A flowchart of the study.

The Guizhou Population Health Cohort Study is a community-based prospective cohort study conducted in Guizhou Province, located in Southwest China. A total of 9,280 adult residents in 48 townships of 12 districts (five urban districts and seven rural counties) in Guizhou Province were enrolled into the cohort between 2010 and 2012 by the multistage proportional stratified cluster sampling method. Eligibility criteria included local residents aged 18 years or older, who had resided in the study area for more than 6 months and had no plans to move out. All participants completed a baseline questionnaire survey, physical examination, and blood sampling. They were followed up from 2016 to 2020 to monitor major chronic diseases and vital status through repeated surveys. Of these, 1,117 participants were lost to follow-up (loss to follow-up rate: 12.04%). The outcome of hypertension was ascertained through questionnaire-based follow-up assessments and physical examinations. A piece of general information: The Death Registration Information System and the Basic Public Health Services System are tasked with confirming the dead state for participants. In the present study, exclusion criteria for participants were as follows: individuals lost to follow-up (*n* = 1,117), those diagnosed with hypertension (*n* = 2,132) or who had missing hypertension data (*n* = 4) or incomplete lifestyle data (*n* = 127) at baseline, and those without blood pressure information (*n* = 406) or had incomplete lifestyle data (*n* = 1,751) at follow-up. Finally, the remaining 3,743 participants were eligible for the analysis (Figure 1). This study was approved by the Institutional Review Board of Guizhou Centre for Disease Control and Prevention (No. S2017-02), and all participants signed a written informed consent form.

### 2.2 Assessment of lifestyle factors and other covariates

All participants received face-to-face interviews conducted by trained investigators through standard procedures. The structured questionnaire was designed by the Chinese Center for Disease Control and Prevention (19, 20) and applied in the China Non-communicable Disease Surveillance in 2010. It includes information on sociodemographic characteristics, lifestyle factors, and medical history. The participants completed this structured questionnaire and provided information relating to sociodemographic characteristics (age, sex, and education level), lifestyle factors (smoking, alcohol intake, sleep status, and sedentary behavior), and family history of chronic disease and personal medical history (hypertension, diabetes mellitus, and dyslipidemia). For ascertaining their dietary habits, information on the frequency and consumption of various food groups in the last few years was obtained by using a semiquantitative food frequency questionnaire. The consumption of edible oil and salt in a family in the recent 30 days was assessed using a family questionnaire, and then the average daily intake of each participant was estimated by dividing the number of people in a family. Never smoking was defined as “never using tobacco products in previous years”; similarly, never drinking was defined as “never consuming alcoholic beverages.”

Anthropometric measurements such as height, weight, and blood pressure were taken using calibrated instruments following standardized protocols. Height and weight were measured with the participants wearing lightweight clothing without shoes and recorded to the nearest measurement of 0.1 cm or 0.1 kg, respectively. Body mass index (BMI) was calculated as weight in kilograms divided by the square of height in meters. Blood pressure was measured three consecutive times at 1-min intervals from the left arm after the participants had rested in a seated position for at least 5 min. The average of the recorded systolic blood pressure (SBP) and diastolic blood pressure (DBP) value was calculated as each individual's blood pressure measurement. Venous blood samples were provided to measure fasting plasma glucose (FPG), high-density lipoprotein cholesterol (HDL-C), low-density lipoprotein cholesterol (LDL-C), total cholesterol (TC), and triglyceride (TG) levels for at least a 8 h fasting period from whole participants.

### 2.3 Definition of HLS

According to the recommendations of Chinese dietary guidelines (21) and previous studies (22–24), the HLS was constructed based on nine lifestyle factors: never smoking, never drinking, sedentary time <6 h/day, sleeping time 7–9 h/day, normal BMI (18.5–23.9 kg/m^2^), vegetable intake ≥300 g/day, fruit intake ≥200 g/day, edible oil intake ≤25 g/day, and salt intake ≤6 g/day. For each healthy lifestyle factor, the participants received 1 point if they met the predefined healthy criteria. Higher scores indicated healthier lifestyle patterns compared with lower scores. In order to ensure that the sample distribution was reasonable and each group had enough participants, the four lower categories (scores 0–3) were merged into one group, while those with a score of ≥6 were grouped separately. Therefore, the HLS was divided into four groups (0–3, 4, 5, and ≥6).

### 2.4 Diagnostic criteria

Hypertension was defined as one of the following conditions: (a) SBP ≥140 mmHg and/or DBP ≥90 mmHg and (b) self-reported diagnosis of hypertension or receiving hypertension treatment. Dyslipidemia was defined as one of the following conditions: (a) TC ≥5.2 mmol/L, (b) TG ≥1.7 mmol/L, (c) HDL-C <1.0 mmol/L, (d) LDL-C ≥3.4 mmol/L, and (e) having a self-reported history of dyslipidemia or current use of lipid-lowering medications.

### 2.5 Statistical analysis

Continuous variables were presented as means and standard deviations, while categorical variables were expressed as frequencies and percentages. Differences in baseline characteristics of the participants with or without hypertension of follow-up were compared by using Student's t-test for continuous variables or the Chi-square test for categorical variables, respectively. Person-years (PYs) were computed as the interval between the completion of baseline investigation and the diagnosis of hypertension and from the date for mortality or completion of the latest follow-up interview, whichever came first.

Unadjusted and multivariable-adjusted Cox proportional hazards regression models were used to estimate the hazard ratios (HRs) and corresponding 95% confidence intervals (CIs) for the association between HLS and the incidence of hypertension. Furthermore, the associations between long-term changes in HLS and the risk of hypertension were examined. Several variables were adjusted in the multivariable model, such as age (as continuous), gender (male or female), area (urban or rural), educational level (primary school or below, junior or senior high school, and college or above), dyslipidemia (yes or no), hypertension family history (yes or no), and FBG (as continuous) at baseline. The QR method can be used to compare the total distribution of a continuous dependent variable or a specific quantile of the dependent variable across diverse groups. The advantage of QR is that it allows for the examination of relationships between levels of exposure and the dependent variable at any point in its distribution, rather than focusing solely on the mean. In this study, QR was applied to estimate the regression coefficients for HLS across a range of blood pressure levels from the 0.1 to the 0.9 quantile, to determine whether the effects of HLS vary across different levels of blood pressure.

Three sensitivity analyses were conducted to assess the robustness of the results: (a) excluding new cases of hypertension were diagnosed within 2 years of follow-up, (b) excluding participants with diabetes at baseline, and (c) adjusting for the cutoff value of salt intake from 6 g/day to 5 g/day. Schoenfeld residuals were used to test the proportional hazards assumption of the Cox regression models, and no significant violation of the proportionality assumption was observed (*P* ≥ 0.05). A two-sided *P*-value < 0.05 was considered statistically significant. All analyses were conducted using R version 3.6.0.

## 3 Results

### 3.1 Baseline characteristics of the participants

The distribution of the baseline characteristics according to the follow-up hypertension status is presented in Table 1. At baseline, the average age of all participants was 42.36 ± 13.40 years. Among them, 1,686 (45.04%) were males, and 1,189 (31.77%) resided in urban areas. Compared with the participants free of hypertension, newly diagnosed hypertension cases significantly tended to contain older patients, constituted a higher proportion of males, and comprised of those with lower levels of education. Among the new-onset hypertension cases, the proportion of those who never smoked, had appropriate sleep time, maintained a normal BMI, met the intake standards for edible oil and salt, reported family history of hypertension, or had a high HLS was lower; in contrast, their baseline SBP and DBP levels were higher. No significant differences were observed for other variables.

**Table 1:** Baseline characteristics according to the follow-up hypertension status.

Characteristic	Total (*n* = 3,743)	Hypertension (*n* = 857)	Without hypertension at follow-up (*n* = 2,886)	*p*-value
Age (years)	42.36 ± 13.40	47.58 ± 14.12	40.81 ± 13.58	<0.001
Gender				0.004
Male	1,686 (45.04)	423 (49.36)	1,263 (43.76)	
Female	2,057 (54.96)	434 (50.64)	1,623 (56.24)	
Area				0.491
Urban	1,189 (31.77)	264 (30.81)	925 (32.05)	
Rural	2,554 (68.23)	593 (69.19)	1,961 (67.95)	
Educational level				<0.001
Primary school or below	1,992 (53.22)	537 (62.66)	1,455 (50.42)	
Junior or senior high school	1,565 (41.81)	290 (33.84)	1,275 (44.18)	
College or above	186 (4.97)	30 (3.50)	156 (5.41)	
Never smoking	2,704 (72.24)	589 (68.73)	2,115 (73.28)	0.009
Never drinking	2,535 (67.73)	582 (67.91)	1,953 (67.67)	0.895
Sleeping time 7–9 h/day	2,274 (60.75)	482 (56.24)	1,792 (62.09)	0.002
Sedentary time <6 h/day	3,030 (80.95)	708 (82.61)	2,322 (80.46)	0.158
Normal BMI (18.5–23.9 kg/m^2^)	2,487 (66.44)	544 (63.48)	1,943 (67.33)	0.036
Vegetable intake ≥300 g/day	2,518 (67.27)	565 (65.93)	1,953 (67.67)	0.339
Fruit intake ≥200 g/day	233 (6.22)	44 (5.13)	189 (6.55)	0.132
Edible oil intake ≤25 g/day	846 (22.60)	156 (18.20)	690 (23.91)	<0.001
Salt intake ≤6 g/day.	1,086 (29.01)	215 (25.09)	871 (30.18)	0.004
Dyslipidemia	2,170 (57.97)	479 (55.89)	1,691 (58.59)	0.160
Hypertension family history	382 (10.21)	70 (8.17)	312 (10.81)	0.017
FBG (mmol/L)	5.22 ± 1.25	5.24 ± 1.50	5.210 ± 1.160	0.599
SBP (mm Hg)	116.63 ± 11.89	119.66 ± 11.80	115.73 ± 11.77	<0.001
DBP (mm Hg)	73.89 ± 7.91	75.36 ± 7.55	73.46 ± 7.96	<0.001
HLS				<0.001
0–3	715 (19.10)	198 (23.10)	517 (17.91)	
4	894 (23.88)	211 (24.62)	683 (23.67)	
5	977 (26.10)	233 (27.19)	744 (25.78)	
≥6	1,157 (30.91)	215 (25.09)	942 (32.64)	

BMI, body mass index. FPG, fasting plasma glucose. SBP, systolic blood pressure. DBP, diastolic blood pressure. HLS, healthy lifestyle scores.

### 3.2 Association of HLS with incidence of hypertension

**Figure 2:**
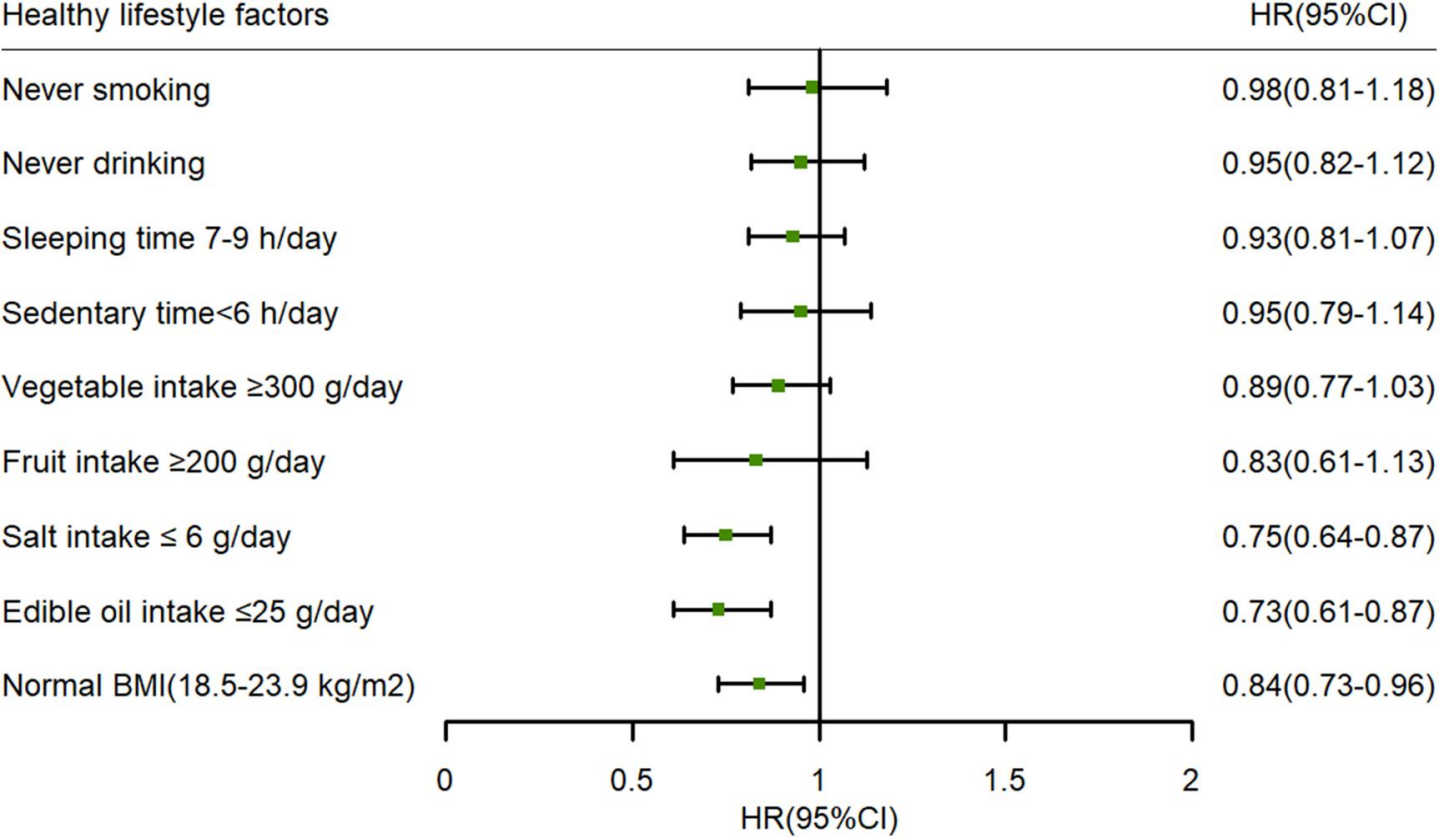
The influence of each healthy lifestyle factor on the risk of hypertension: adjusted age (as continuous), gender (male or female), area (urban or rural), education level (primary school or below, junior or senior high school, and college or above), dyslipidemia (yes or no), hypertension family history (yes or no), and FBG (as continuous) at baseline. BMI, body mass index; HR, hazard ratio; 95% CI, 95% confidence interval; FPG, fasting plasma glucose.

The influence of each healthy lifestyle factor on the risk of hypertension is described in Figure 2. In this study, optimal BMI and low intake of edible oil and salt were associated with a negative correlation with hypertension. During 25,682.98 PYs of follow-up (median 6.46 years), 857 hypertension cases were identified with an incidence of 33.37/1,000 PYs. The HLS had a protective effect against hypertension (HR: 0.86, 95% CI: 0.80–0.92), with a 14% reduction in hypertension incidence for a 1-unit increase in the HLS. Compared with participants with a HLS of 0–3, the multivariable-adjusted HRs (95% CIs) for participants with HLS of 4, 5, and ≥6 were 0.80 (95% CI: 0.66–0.97), 0.78 (95% CI: 0.64–0.95), and 0.61 (95% CI: 0.49–0.75), respectively, showing a decreasing trend (P for trend <0.001) (Table 2).

**Table 2:** Association between HLS and the risk of hypertension.

HLS	cases	PYs of follow-up	incident density (/1,000 PYs)	HR (95% CI)	
				Model 1	Model 2
Total	857	25,682.98	33.37		
0–3	198	4,732.47	41.84	Reference	Reference
4	211	6,089.80	34.65	0.78 (0.64–0.94)*	0.80 (0.66–0.97)*
5	233	6,741.10	34.56	0.74 (0.61–0.89)*	0.78 (0.64–0.95)*
≥6	215	8,119.61	26.48	0.53 (0.44–0.64)*	0.61 (0.49–0.75)*
P for trend				<0.001	<0.001
HLS (per 1 unit)				0.82 (0.77–0.87)*	0.86 (0.80–0.92)*

Model 1 was unadjusted. Model 2 was adjusted for age (as continuous), gender (male or female), area (urban or rural), education level (primary school or below, junior or senior high school, and college or above), dyslipidemia (yes or no), hypertension family history (yes or no), and FBG (as continuous) at baseline.

PYs, person-years. HR, hazard ratio. 95% CI, 95% confidence interval. HLS, healthy lifestyle scores. FPG, fasting plasma glucose.

* *P* < 0.05.

### 3.3 Associations of long-term changes in HLS with incidence of hypertension

**Figure 3:**
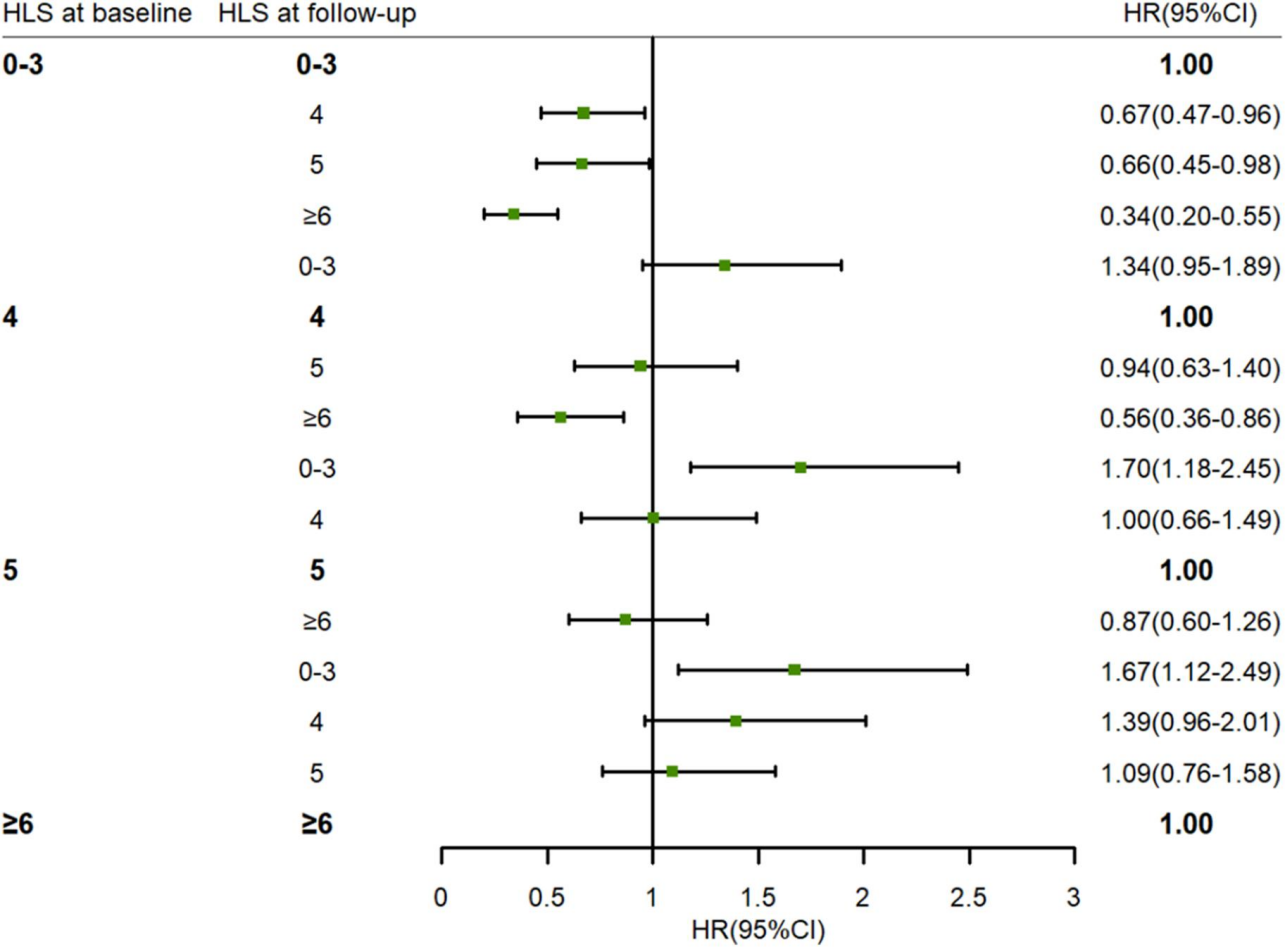
The association between changes in HLS from baseline to follow-up and risk of hypertension. Adjusted age (as continuous), gender (male or female), area (urban or rural), education level (primary school or below, junior or senior high school, college or above), dyslipidemia (yes or no), hypertension family history (yes or no), and FBG (as continuous) at baseline. HLS, healthy lifestyle scores. HR, hazard ratio. 95% CI, 95% confidence interval. FPG, fasting plasma glucose.

As shown in Table 3, long-term changes in HLS were found to be associated with a reduced risk of incident hypertension. In the fully adjusted model, the HR was 0.71 (95% CI: 0.62–0.80) for hypertension with per unit increase in HLS. Compared with participants who maintained a HLS of 0–3, those who maintained HLS of 4 (HR: 0.60, 95% CI: 0.43–0.85), 5 (HR: 0.47, 95% CI: 0.32–0.69), and ≥6 (HR: 0.34, 95% CI: 0.23–0.51) had a lower risk of incident hypertension (P for trend <0.001). Figure 3 shows the associations between changes in HLS from baseline to follow-up and the risk of hypertension, with participants who maintained different HLS levels used as reference groups. An inverse association was observed between increases in HLS during follow-up and the risk of incident hypertension among participants who maintained a HLS of 0–3 or 4. In contrast, participants whose HLS decreased to 0–3 had a higher risk of incident hypertension compared with those who maintained a HLS of 5 (HR: 1.70, 95% CI: 1.18–2.45) or ≥6 (HR: 1.67, 95% CI: 1.12–2.49).

**Table 3:** Associations of long-term changes in HLS with incidence of hypertension.

HLS at baseline	HLS at follow-up	Cases	PYs of follow-up	Incident density (/1,000 PYs)	HR (95% CI)	
					Model 1	Model 2
0–3	0–3	91	1,706.98	53.31	Reference	Reference
4	4	57	1,615.13	35.29	0.59 (0.43–0.83)*	0.60 (0.43–0.85)*
5	5	52	1,711.73	30.38	0.46 (0.32–0.64)*	0.47 (0.32–0.69)*
≥6	≥6	65	2,790.69	23.29	0.32 (0.23–0.44)*	0.34 (0.23–0.51)*
P for trend					<0.001	<0.001
HLS (per 1 unit)					0.69 (0.62–0.76)*	0.71 (0.62–0.80)*

Model 1 was unadjusted. Model 2 was adjusted for age (as continuous), gender (male or female), area (urban or rural), education level (primary school or below, junior or senior high school, and college or above), dyslipidemia (yes or no), hypertension family history (yes or no), and FBG (as continuous) at baseline. PYs, person-years. HR, hazard ratio. 95% CI, 95% confidence interval. HLS, healthy lifestyle scores. FPG, fasting plasma glucose.

* *P* < 0.05.

### 3.4 Associations between HLS and blood pressure levels

**Figure 4:**
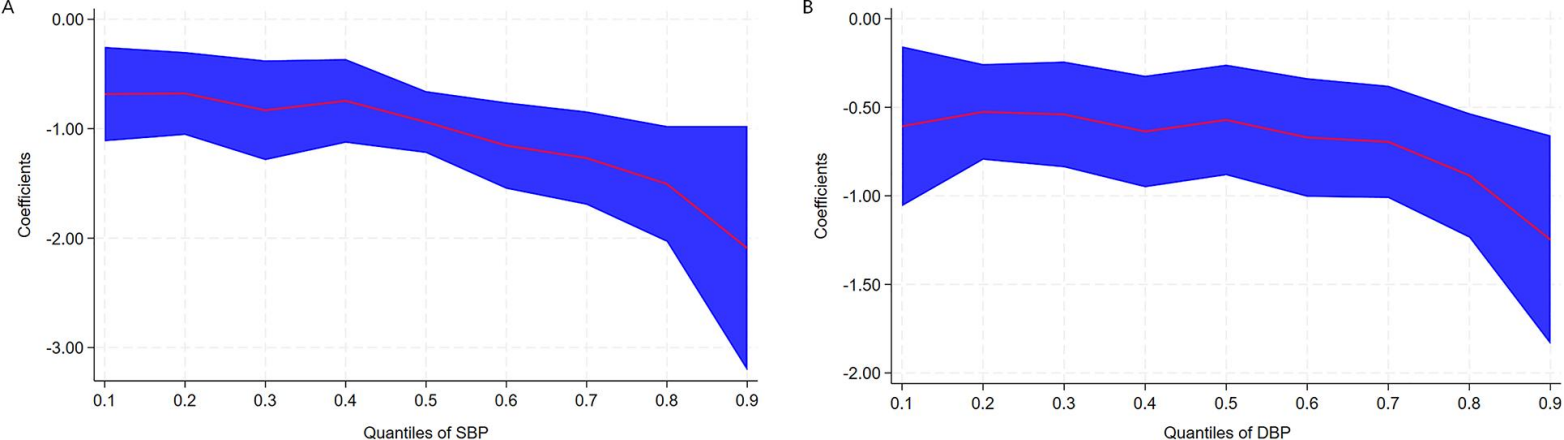
Coefficients (β) for the associations of SBP **(A)** and DBP **(B)** with HLS across the quantile levels of blood pressure. The coefficients indicate the changes in blood pressure levels (mmgH) with a 1-unit (1 score) increase of HLS. Adjusted age (as continuous), gender (male or female), area (urban or rural), education level (primary school or below, junior or senior high school, and college or above), dyslipidemia (yes or no), hypertension family history (yes or no), and FBG (as continuous) at baseline. The red line represents parameter estimates at different QRs, and the blue area represents confidence intervals of the parameter estimates. SBP, systolic blood pressure. DBP, diastolic blood pressure. FBG, fasting plasma glucose. QR, quantile regression.

The coefficient estimates and corresponding 95% CIs for the associations of HLS with the quantile levels of blood pressure are presented in Table 4 and Figure 4. Inverse associations were observed between baseline HLS and both SBP (*β* = −1.07, 95% CI: −1.50, −0.65) and DBP (*β* = −0.76, 95% CI: −1.01, −0.50) during follow-up according to multiple linear regression analyses. Similarly, the results of QR showed that the higher the blood pressure level, the stronger the protective effect of HLS. With a 1-unit increase in HLS at the quantiles of 0.4 and 0.9, SBP decreased by 0.75 mm/Hg (*β* = −0.75, 95% CI: −1.33, −0.16) and 2.09 mm/Hg (*β* = −2.09, 95% CI: −2.79, −1.39). A significant protective effect of HLS on reducing DBP was also observed across quantile levels ranging from 0.5 to 0.9. With a 1-unit increase in HLS at the quantiles of 0.5 and 0.9, DBP decreased by 0.57 mm/Hg (*β* = −0.57, 95% CI: −0.82, −0.31) and 1.25 mm/Hg (*β* = −1.25, 95% CI: −1.82, −0.68).

**Table 4:** Associations between blood pressure levels and HLS at the mean and selected quantile levels of blood pressure.

Quantile levels of blood pressure	SBP		DBP	
	Mean value or corresponding value at the quantile levels (mmHg)	Coefficients (95% CI)^a^	Mean value or corresponding value at the quantile levels (mmHg)	Coefficients (95% CI)^a^
OLS	127.18	−1.07 (−1.50,−0.65)	78.05	−0.76 (−1.01,−0.50)
QR				
0.1	108.00	−0.68 (−1.32,−0.05)	66.00	−0.61 (−0.96,−0.26)
0.2	114.00	−0.68 (−1.44,−0.86)	70.00	−0.53 (−0.78,−0.27)
0.3	118.67	−0.83 (−1.36,−0.30)	72.67	−0.54 (−0.76,−0.32)
0.4	122.00	−0.75 (−1.33,−0.16)	75.33	−0.64 (−0.90,−0.37)
0.5	125.33	−0.94 (−1.35,−0.52)	78.00	−0.57 (−0.83,−0.31)
0.6	129.00	−1.15 (−1.59,−0.71)	80.00	−0.67 (−0.94,−0.40)
0.7	132.33	−1.27 (−1.69,−0.84)	82.00	−0.69 (−1.06,−0.33)
0.8	138.00	−1.50 (−1.97,−1.04)	85.33	−0.88 (−1.28,−0.48)
0.9	148.00	−2.09 (−2.79,−1.39)	90.33	−1.25 (−1.82,−0.68)

SBP, systolic blood pressure; DBP, diastolic blood pressure; HLS, healthy lifestyle scores; OLS, ordinary least squares; QR, quantile regression; 95% CI, 95% confidence interval; FPG, fasting plasma glucose.

a The changes in blood pressure levels (mmHg) with a 1-unit (1 score) increase of HLS. Adjusted age (as continuous), gender (male or female), area (urban or rural), education level (primary school or below, junior or senior high school, and college or above), dyslipidemia (yes or no), hypertension family history (yes or no), and FBG (as continuous) at baseline.

### 3.5 Sensitivity analysis

In this study, a sensitivity analysis was conducted, the results of which did not differ substantially from those of the primary analysis.

## 4 Discussion

In this study, we comprehensively analyzed the associations of individual healthy lifestyle factors and overall HLS with the risk of hypertension, based on a prospective cohort study conducted in Southwest China. The results indicated that among the various individual healthy lifestyle factors, optimal BMI and appropriate intake of edible oil and salt were protective factors against hypertension. In addition, a strong inverse correlation between HLS and the risk of hypertension in adults was observed. Particularly, with longitudinal assessments of HLS at both baseline and follow-up, this study demonstrated a link between adherence to a higher HLS and a significant reduction in the risk of incident hypertension. In contrast, a decrease in HLS was associated with an increased risk of hypertension.

In this study, we found that several favorable lifestyle factors, including optimal BMI and appropriate intake of edible oil and salt, were inversely associated with hypertension. In existing studies, BMI has been consistently identified as a significant factor influencing hypertension (25, 26). These studies, including the present study, have demonstrated the important role of optimal body weight in hypertension prevention. In this study, we found that there was a distinct profitable association between less consumption of oil and salt and hypertension in our research, and the results were consistent with those of the many previous studies (27–29). In actual terms, the average intake of edible oil and salt in Chinese adults was markedly higher than the upper limit of 2016 Chinese Diet Pagoda (30). Given this, reducing oil and salt consumption is important for delaying the occurrence of hypertension.

In the present study, several relatively modifiable lifestyle factors were combined to evaluate their impact on hypertension. Although only a few healthy lifestyle factors are associated with hypertension, our findings confirmed that the risk of hypertension gradually decreased with the increase of HLS, which are consistent with those of previous studies, despite minor variations in the specific health lifestyle factors examined (31–33). The possible reason was that a certain healthy lifestyle factor had no significant effect on hypertension, but the synergistic effect of specific lifestyle combinations appeared to decrease the risk of hypertension. A large prospective cohort study conducted in France examined the combined impact of four lifestyle factors on the incidence of hypertension. The results revealed that participants adhering to all these healthy lifestyles could halve the risk of hypertension (34). The SUN cohort study found that a healthy lifestyle pattern constructed from six healthy habits was linearly associated with a decrease risk of hypertension, and participants with the highest healthy lifestyle scores exhibited a 46% relative reduction in the risk of developing hypertension compared with those with the lowest scores (25). Another prospective cohort study from the UK Biobank found that both higher HLS and healthy sleep pattern were associated with a reduction of hypertension risk (35). In contrast, the result of a cross-sectional analysis from a large cohort study indicated that a combination of several unhealthy lifestyle factors was strongly associated with risk of hypertension (36). Moreover, our study found that HLS was strongly and inversely associated with blood pressure values, which is comparable to the previous findings (14, 37). However, our results exhibit a relatively high effect magnitude, especially at high quantiles of blood pressure, whether for SBP or for DBP. One contributing factor lies in the variations of participant selection criteria across surveys. The aforementioned studies primarily focused on middle-aged and older adults, who were generally in poorer physical condition compared with younger groups, and blood pressure management also tends to be more challenging within this demographic. Our results add to the existing evidence that maintaining a healthy lifestyle helps prevent and control the development of hypertension, especially for those with elevated blood pressure levels, and the benefits of a healthy lifestyle are obvious.

Previous studies have demonstrated the impact of changes in lifestyle factors on the risk of incident hypertension. Two cohort studies from China indicated that compared with stable overweight and obese status, subsequent weight loss exhibited a significantly 33% and 40% lower risk of hypertension, respectively (38, 39). A large cohort study conducted in Korea discovered that both baseline physical activity level and its temporal change were associated with the risk of hypertension (40). Another study also found that individuals with substantial changes in sleep duration were at greater risk of developing hypertension (41). Although these studies have identified the influence of changes in certain lifestyle factors on hypertension, little is known about the health effects associated with changes in overall lifestyle habits. Our study further explored the impact of changes in HLS on the risk of incident hypertension. On the one hand, we found that great benefit could be had from long-term adherence to a high HLS compared with maintaining a lower HLS. A prospective study involving 7,671 Chinese individuals showed that the risk of hypertension in a group that consistently had a HLS >3 was 0.61 times that of a group that maintained a HLS of 0–2 (37). Another cohort study from China found that a high stable trajectory of HLS was inversely related to a reduced risk of CVD risk compared with a low stable trajectory (42). Since hypertension is an important risk factor for CVD, this finding supports the notion that long-term maintenance of a high HLS is beneficial for blood pressure health in the Chinese population. On the other hand, we observed that participants who improved their HLS from 0 to 3 to 4 or more had a lower risk of incident hypertension than those who maintained a HLS of 0–3; in other words, the higher the HLS, the lower the risk of hypertension. In contrast, participants with a reduced HLS of 3 or less were found to have an increased risk of hypertension compared with those with consistently high HLS. Given that a large proportion of the population modify their lifestyle over time (43), these findings highlight the importance of sustaining a healthy lifestyle and improving from an unhealthy to a healthy lifestyle over the long term.

This study has several strengths, including the prospective study design, the relatively long follow-up period, stringent quality control procedures, and the high volume of information about lifestyle and risk factors. Nevertheless, several limitations of our study should be considered when interpreting the results. First, even though we used the standardized questionnaire and lifestyle factors were assessed through self-reports, some recall biases and measurement errors were inevitable. Second, there were no adjustments for other lifestyle factors such as use of antihypertensive medication and overall energy intake. Third, the timing of HLS changes and the influence of HLS duration on hypertension remain unclear. Finally, the participants in this study were permanent residents of Guizhou Province, China, and therefore, the results may not be generalizable to other populations.

In conclusion, higher levels of HLS are associated with a progressively low risk of hypertension, particularly among individuals with elevated blood pressure. Moreover, long-term maintenance of a high HLS would further reduce the likelihood of the occurrence of hypertension. Our findings highlight the importance of sustaining a healthy lifestyle throughout one’s life and promptly modifying unhealthy habits to prevent hypertension.

## Data Availability

The datasets for this manuscript will be made available upon request, further inquiries can be directed to the corresponding author.
